# Ultrasonic-induced transalkylation of heavy alkylbenzene to linear alkylbenzene using aluminum chloride: A green and efficient pathway for sustainable detergent production

**DOI:** 10.1016/j.ultsonch.2025.107648

**Published:** 2025-10-23

**Authors:** Mosaad A. Kasaby, Wagih A. Sadik, Abdallah S. Elgharbawy, Ahmed A. Ghazy

**Affiliations:** a Materials Science Department, Institute of Graduate Studies and Research (IGSR), Alexandria University, 163 Horrya Avenue, P.O. Box 832, Shatby 21526 Alexandria, Egypt; b The Egyptian Ethylene and Derivatives Company (Ethydco), Alexandria, Egypt

**Keywords:** Ultrasonic-induced transalkylation, Linear Alkylbenzene (LAB), Heavy Alkylbenzene (HAB), Aluminum chloride catalyst (AlCl_3_), Green chemistry

## Abstract

The efficient production of linear alkylbenzene (LAB) from heavy alkylbenzene (HAB) has become increasingly essential due to the global demand for highly efficient detergents. This study introduces an innovative ultrasonic-induced transalkylation process, leveraging HAB as a catalyst to enhance reaction efficiency while ensuring sustainability. Unlike traditional methods, which often suffer from high energy consumption, prolonged reaction times, and environmental drawbacks, the proposed technique harnesses ultrasonic energy to significantly accelerate molecular interactions, reducing reaction time and operating temperatures without compromising product quality. Our comprehensive experimental analysis revealed that ultrasonic waves, combined with aluminum chloride (AlCl_3_) catalysis, achieved a significant conversion efficiency of HAB to LAB. Notably, this method outperformed conventional catalytic approaches by producing LAB with high purity, viscosity, and density that meet industrial standards. This ultrasonic-induced process presents a sustainable, cost-effective, and scalable alternative for the large-scale production of LAB, offering significant environmental and operational advantages over traditional methods.

## 1 Introduction

LAB is a cost-effective and biodegradable compound widely used in the production of detergents and surfactants. The significance of LAB production has increased substantially in response to the global rising demand for detergents, particularly during public health crises such as the COVID-19 pandemic [1]. LAB is the primary raw material in detergent manufacturing, and its synthesis typically involves the alkylation of benzene with linear olefins through the Friedel-Crafts reaction. Although homogeneous acid catalysts such as hydrofluoric acid and HAB are effective in this process, they pose significant environmental and operational challenges, including equipment corrosion and the generation of hazardous waste [2]. To address these limitations, solid acid catalysts have emerged as promising alternatives, offering reduced environmental impact and enhanced reusability, though with challenges in recovery and regeneration.

Historically, dodecylbenzenes were widely used in detergent formulations due to their efficacy; however, their non-biodegradable nature led to long-term environmental pollution, including pollution of water bodies and marine ecosystems [3]. In response, the 1960s experienced a paradigm shift towards the use of alkylbenzene sulfonates, driven by their superior biodegradability. Countries across Europe, Japan, and the United States led this transition, replacing dodecylbenzene sulfonates with linear alkylbenzene sulfonates (LABS) [4,5]. The adoption of LABS, derived from LAB, has since become the industry standard, due to its cost-efficiency, enhanced biodegradability, and superior environmental profile. LAB technology, which involves converting normal paraffins to olefins followed by benzene alkylation, has largely replaced branched alkylbenzene technologies [6,7].

The production of LAB generates HAB as a by-product that primarily is used in specialized applications such as lubricating greases and heat-transfer fluids, presents several drawbacks [8]. Its higher molecular weight and lower biodegradability compared to LAB make it less suitable for detergent applications, posing significant environmental concerns. HAB also accumulates within industrial equipment, causing operational inefficiencies and increased maintenance requirements [9]. HAB is a pivotal catalyst in modern industrial chemistry, known for its strong Lewis acidity and versatility in catalyzing a wide range of reactions [10].

The sulfonation products of HAB have weaker surfactant properties compared to those derived from LAB, further limiting their utility. As a result, industries have transitioned mainly to LAB for surfactant and detergent applications, recognizing its superior performance and reduced ecological footprint. Structurally, LAB comprises hydrocarbon chains with a benzene ring spanning C10 to C13, while HAB contains longer chains from C14 to C28, contributing to their distinct physicochemical and environmental properties [6,11].

Aluminum chloride (AlCl_3_) plays a central role in Friedel-Crafts alkylation, facilitating the formation of reactive carbocations and enabling high selectivity in product formation [12,13]. Its continuous relevance in industrial applications is highlighted by its efficiency in alkylation processes and ease of separation from reactants. The reaction pathways mediated by AlCl_3_ have been extensively studied, emphasizing the compound's capacity to isomerize primary alkyl halides and generate unique isomeric derivatives[14]. AlCl_3_ exhibits higher catalytic activity under milder reaction conditions than solid acid catalysts such as zeolites, which results in increased transalkylation efficiency [15]. This study seeks to build upon these advancements by integrating ultrasonic energy with AlCl_3_ catalysis, aiming to enhance efficiency and sustainability in the conversion of HAB to LAB.

Ultrasound is essential for improving transalkylation reactions because it creates cavitation effects, which provide localized high-temperature and high-pressure microenvironments. By promoting bond cleavage and reformation, these harsh circumstances enhance conversion efficiency and reaction kinetics. In addition, ultrasonic waves improve mass transfer between catalysts and reactants, which improves catalyst dispersion and boosts catalytic efficiency [16]. In transalkylation, ultrasound facilitates the dispersion of AlCl_3_, enhances the accessibility of reactive sites on HAB, and accelerates reaction kinetics, enabling efficient conversion to LAB under milder conditions compared to traditional methods [17,18].

Ghadiri et al. [9] developed green catalysts, including sulfuric acid-activated bentonite clay, alumina-pillared bentonite clay, and alumina-pillared sulfuric acid-activated bentonite clay, for transalkylation of heavy alkylbenzenes (HABs) with benzene to produce monoalkylbenzenes. These catalysts were characterized using X-ray diffraction, Fourier transform infrared spectroscopy, nitrogen adsorption–desorption isotherms, and temperature-programmed desorption of NH_3_. The batch slurry reactor achieved optimal performance at 225 °C and a benzene/HAB ratio of 16, resulting in an 80 % HAB conversion rate. Tsai et al. [19] investigated benzene and xylene production via transalkylation of HABs using mordenite catalysts. Base-treated mordenite (BaseM7) exhibited superior catalytic stability and performance over 100 h. Using a 50:50 feed ratio of toluene and 1,2,4-trimethylbenzene (TMB) in a fixed-bed continuous reactor at 553 K, 2068 kPa, a weight hourly space velocity (WHSV) of 3.0 h^−1^, and a hydrogen-to-feed ratio of 3.0 mol/mol, BaseM7 achieved a 42.8 % yield of benzene and xylene with 100 % benzene purity. The base treatment enhanced mesopores (up to 7 nm) and reduced coke formation, improving energy efficiency and environmental impact.

Tsai et al.[20] further studied toluene transalkylation with 1,3,5-trimethylbenzene over Pt/mordenite and Pt/ZSM-12 catalysts. Reactions at 673 K, 21.4 kg/cm^2^, WHSV of 5.5 h^−1^, and a hydrogen-to-hydrocarbon molar ratio of 3.0 demonstrated the effectiveness of Pt/mordenite in achieving high catalytic stability and benzene purity (up to 39.3 % A6 + A8). Sulfurization of Pt/ZSM-12 yielded benzene with 93.5 % purity alongside high xylene yields, highlighting the importance of controlled hydrogenation activity for product purity and stability. Al-Zahrani et al.[21] investigated the kinetics and mechanisms of transalkylation, disproportionation, and isomerization of *meta*-diethylbenzene (m-DEB) in the presence of benzene using triflic acid as a catalyst. Reactions were carried out in a liquid-phase batch reactor at atmospheric pressure, and at temperatures between 288 and 308 K, under continuous stirring in a nitrogen atmosphere. It included only primary reactions, and the main product was ethylbenzene; triethylbenzene and isomers of DEB were by-products. The yield measurements after 4 h gave an EB yield of 24.2 % at 298 K.

This study introduces an innovative ultrasonic-induced transalkylation process that represents a paradigm shift in HAB-to-LAB conversion. By integrating ultrasonic energy with AlCl_3_ catalysis, this approach leverages cavitation phenomena to accelerate molecular interactions, reduce energy barriers, and enhance reaction kinetics. The primary objective of this research is to optimize the key parameters influencing ultrasonic-induced transalkylation, including ultrasonic. Unlike traditional methods that rely on high temperatures, severe pressures, and prolonged reaction times, this approach utilizes ultrasonic cavitation to enhance reaction kinetics, achieving high LAB purity (over 91 % C10–C13 isomers) in just 5 min at 20 °C. This method aligns with green chemistry principles by reducing energy consumption, minimizing side reactions, and enabling catalyst recovery through simple filtration, thus offering a sustainable and scalable alternative for industrial LAB production.

## 2 Materials and methods

### 2.1 Materials

Benzene with a molecular weight (Mw) of approximately 78.11 g/mol and purity of 98 % was purchased from El Naser Pharmaceutical Chemical Company. LAB with a molecular weight (Mw) of approximately 242 g/mol with a purity of 98 % and HAB with a molecular weight (Mw) of approximately 357.7 g/mol were supplied by Egyptian Linear Alkylbenzene Company (ELAB). AlCl_3_ with a molecular weight (Mw) of approximately 133.34 g/mol was purchased from the El Naser Pharmaceutical Chemical Company.

### 2.2 Methods

**Fig. 1:**
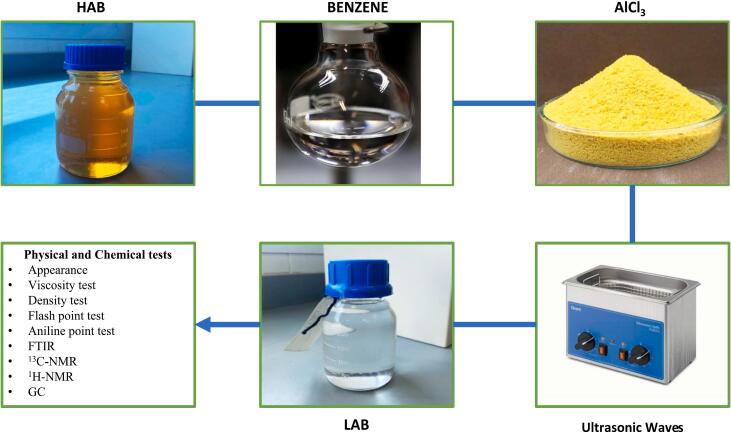
Diagram of the ultrasonic-assisted transalkylation of HAB to LAB using AlCl_3_ catalyst.

HAB, benzene, and AlCl_3_ were subjected to ultrasonic treatment using an Elmasonic Easy 50 R ultrasonic bath (Elma Schmidbauer GmbH, Germany) operating at a fixed frequency of 37 kHz, with an effective ultrasonic power of 150 W under ambient air conditions. Fig. 1 shows the sequential steps of the ultrasonic-assisted conversion of heavy alkylbenzene (HAB) into linear alkylbenzene (LAB) using aluminum chloride (AlCl_3_) as a Lewis acid catalyst. The method started by reaction the HAB molecules with benzen by ultrasonic cavitation, increasing mass transfer and allowing the process of catalytic transalkylation, thus making the production of LAB with high selectivity. The resulting LAB was further analyzed through a detailed set of physicochemical characterization experiments in order to evaluate the quality. The analyses comprised appearance, kinematic viscosity, specific gravity, flash point, aniline point, and structural verification through Fourier-transform infrared (FTIR) spectroscopy, ^13^C nuclear magnetic resonance (^13^C NMR), ^1^H nuclear magnetic resonance (^1^H NMR), and gas chromatography (GC).

#### 2.2.1 Experimental procedure

The transalkylation reaction was conducted in a 100 mL round-bottom flask containing 10 g of HAB (Mw 357.7 g/mol, 98 % purity, ELAB), benzene (Mw 78.11 g/mol, 98 % purity, El Naser Pharmaceutical Chemical Company) at various molar ratios, and AlCl_3_ (Mw 133.34 g/mol, 98 % purity, El Naser) at concentrations ranging from 1 to 10 wt%. The reaction mixture was stirred at 300 rpm using a magnetic stirrer to ensure uniform mixing. Ultrasonic irradiation was applied using an Elmasonic Easy 50 R ultrasonic bath (Elma Schmidbauer GmbH, Germany) operating at a fixed frequency of 37 kHz, with an effective power of 150 W. The reaction vessel was positioned centrally in the ultrasonic bath, filled with distilled water as the coupling medium, and reactions were conducted at temperatures ranging from 20 to 80 °C for durations of 5 to 20 min under ambient air conditions. After the reaction completion, the AlCl_3_ catalyst was recovered via simple filtration using a Buchner funnel with Whatman No. 1 filter paper, and the liquid product was collected for analyses.

Table 1 summarizes the influence of key parameters on the ultrasonic-induced conversion of HAB to LAB. **Time Module**: The effect of ultrasonic treatment duration (5–20 min) on conversion efficiency was evaluated at a constant temperature of 20 °C. Each sample contained 10 g of HAB, 10 g of benzene (4:1 M ratio), and 10 % (1 g) AlCl_3_ catalyst. **Benzene-to-HAB Ratio Module**: The impact of varying the benzene-to-HAB molar ratio (1:1 to 10:1) on the conversion rate was examined. All samples contained 10 g of HAB and 10 % (1 g) AlCl_3_ catalyst, with ultrasonic treatment conducted at 20 °C for 5 min. **Catalyst Module**: The role of AlCl_3_ concentration (1–10 wt%) was assessed, with all samples containing 10 g of HAB, a 4:1 benzene-to-HAB ratio, and treated ultrasonically for 5 min at 20 °C. **Temperature Module**: The influence of reaction temperature (20–80 °C) on conversion efficiency was investigated using samples containing 10 g of HAB, a 4:1 benzene-to-HAB ratio, and 10 % (1 g) AlCl_3_ catalyst, with ultrasonic treatment conducted for 5 min.

**Table 1:** Operating conditions for the transalkylation of HAB to LAB.

**Sample code**	**HAB (gm)**	**Benzene to HAB ratio**		**Catalyst**		**Ultrasonic treatment**	
		**Ratio**	**Weight (gm)**	**Concentration %**	**Weight (gm)**	**Time(min)**	**Temperature (^o^C)**
Time module							
HBA01	10	4:1	10	10	1	5	20
HBA02	10	4:1	10	10	1	10	20
HBA03	10	4:1	10	10	1	15	20
HBA04	10	4:1	10	10	1	20	20
Benzene to HAB module							
HBA05	10	1:1	2.5	10	1	5	20
HBA06	10	2:1	5	10	1	5	20
HBA07	10	4:1	10	10	1	5	20
HBA08	10	8:1	20	10	1	5	20
HBA09	10	10:1	25	10	1	5	20
Catalyst module							
HBA10	10	4:1	10	1	0.1	5	20
HBA11	10	4:1	10	2	0.2	5	20
HBA12	10	4:1	10	4	0.4	5	20
HBA13	10	4:1	10	8	0.8	5	20
HBA14	10	4:1	10	10	1	5	20
Temperature module							
HBA15	10	4:1	10	10	1	5	20
HBA16	10	4:1	10	10	1	5	40
HBA17	10	4:1	10	10	1	5	60
HBA18	10	4:1	10	10	1	5	80

#### 2.2.2 Characterization of products and feedstock

##### 2.2.2.1 Quality control and assurance

To ensure reproducibility, all instruments (e.g., viscometer, FTIR, NMR, GC) were calibrated according to manufacturer specifications before each experiment. Samples were analyzed in triplicate, with average values reported. Standard reference materials (e.g., commercial LAB) were used to validate measurements, ensuring accuracy within ± 2 % for viscosity and density, ±1°C for aniline and flash points, and ± 0.5 % for GC peak areas. Benzene and HAB purity (98 %) were verified via GC prior to reactions.

##### 2.2.2.2 Statistical analysis

Statistical Analysis Viscosity and density measurements were conducted in triplicate, and data were analyzed using one-way ANOVA to assess the significance of differences across experimental conditions (time, benzene-to-HAB ratio, catalyst concentration, temperature). A p-value < 0.05 was considered significant, indicating that variations in viscosity and density were statistically significant across the tested parameters.

##### 2.2.2.3 Viscosity and density

The viscosity and density of the samples were measured according to ASTM D445 and ASTM D1298 standards, respectively. ASTM D-445 determines the kinematic viscosity of liquids by measuring the time required for a specified volume to flow through a calibrated glass capillary viscometer under gravity. Samples were homogenized by stirring or by gentle warming to remove air bubbles and solids. The viscometer tube was cleaned and dried before testing at controlled temperatures of 40 °C or 100 °C. Kinematic viscosity (ν) in centistokes (cSt) was calculated using ν = C × t, where t is the flow time in seconds and C is the viscometer constant. Density measurements followed ASTM D1298, which determines the density or relative density of crude oils and petroleum products. Homogeneous, bubble-free samples were prepared with light heating applied to viscous samples as needed. A clear cylinder was filled to allow free hydrometer flotation, and the hydrometer reading was taken at the liquid surface after stabilization. The corresponding temperature was recorded for density correction. Density results, expressed in grams per cubic centimeter (g/cm^3^), provided essential insights into the mass and flow properties of the samples.

##### 2.2.2.4 Aniline point

The aniline point, determined in accordance with ASTM D611, quantifies the aromatic content of hydrocarbon-based materials such as fuels and lubricating oils. It is defined as the lowest temperature at which equal volumes of aniline and the hydrocarbon sample form a homogeneous solution; it serves as an indicator of the material’s aromatic composition and chemical characteristics. The procedure involves mixing equal volumes of the hydrocarbon sample and aniline in a sealed container, followed by heating in a thermostatically controlled enclosure, typically an oil bath, under continuous stirring. The aniline point is recorded as the temperature at which complete miscibility is achieved. For verification, the mixture may be cooled to confirm the reappearance of phase separation. This test provides critical insights into the chemical structure and performance properties of hydrocarbon materials.

##### 2.2.2.5 Flash point

The flash point, as determined by ASTM D93, represents the minimum temperature at which a material generates sufficient vapor to form an ignitable mixture with air in the presence of a flame. This parameter is critical for assessing the flammability and combustion risk of petroleum and chemical substances, playing a vital role in ensuring their safe handling, storage, and transportation. The measurement utilizes the Pensky-Martens Closed Cup (PMCC) method. In this procedure, the sample is placed in a sealed cup apparatus and subjected to controlled heating. A flame is intermittently introduced above the liquid surface, and the temperature at which vapor ignition is visually observed is recorded as the flash point. This method provides precise and reproducible data, enabling the classification of flammable materials and the development of effective fire hazard mitigation strategies.

##### 2.2.2.6 Fourier transform infrared spectroscopy (FTIR)

The functional groups of HAB and the produced samples of LAB were investigated by FTIR in the range of 600–4000 cm^−1^. A solid plate made from potassium bromide (KBr) was covered wholly by the liquid sample, then the plate was inserted into the FTIR device (Bruker Tensor 37).

##### 2.2.2.7 Nuclear magnetic resonance spectroscopy (NMR)

One of the most effective analytical methods for determining molecular structure, especially in organic and polymer chemistry, is nuclear magnetic resonance (NMR) spectroscopy. It provides detailed insights into the electronic environment of a molecule’s nucleus, allowing the identification of various functional groups and their interactions. NMR spectroscopy is based on the interaction of atomic nuclei with an external magnetic field. Nuclear spin is a characteristic of several isotopes, including carbon (13C) and hydrogen (1H). These nuclei absorb energy and change between spin states when subjected to radiofrequency radiation and a strong magnetic field. An NMR spectrum is created from the consequent energy absorption, which provides crucial insights into the chemical structure. Numerous scientific and industrial fields, such as materials science, petrochemicals, and medicines, heavily rely on NMR spectroscopy. It is essential for verifying the structure of synthetic substances, evaluating their purity, and identifying contaminants in intricate combinations. In order to confirm the molecular structure of LAB and assess its chemical environment, NMR spectroscopy was used for structural characterization. Deuterated chloroform (CDCl_3_) was used as the solvent in the analysis, which was carried out using a **JNM-ECZ500R** spectrometer running at 500 MHz for ^1^H NMR and 126 MHz for ^13^C NMR. The effective synthesis of LAB was confirmed by analyzing and comparing the acquired spectra with reference data.

##### 2.2.2.8 Gas chromatography (GC)

Gas chromatography (GC) is the basic analysis method used for the separation and determination of individual hydrocarbons in mixed streams. In LAB production, GC delivers important information about isomer distribution in the product and allows confirmation of the efficiency and selectivity of the conversion of HAB to LAB. The presence of a high concentration of LAB-range isomers, which are generally considered C10–C13, and trace quantities of non-LAB materials signifies a successful transformation. In this study, GC analysis was performed using a **Bruker Scion 436-GC** gas chromatograph.

## 3 Result and discussion

A thorough series of experimental investigations, including measurements of viscosity, density, aniline point, and flash point, is included in this paper, along with visual examinations to confirm the findings.

### 3.1 Raw materials properties

Table 2 presents a comparative analysis of the viscosity and density of LAB, Benzene, and HAB. The kinematic viscosity values, measured at 40 °C, reveal significant differences in flow behavior among the materials. Moreover, the differences in density values measured at 15 °C enhance the ability to distinguish between the materials. These standardized measurements underscore the unique rheological and mass properties of each substance, reflecting their underlying compositional and structural differences.

**Table 2:** Viscosity and density values of raw materials.

**Raw material**	**Viscosity At 40◦ C(cSt)**	**Density at 15^◦^C (g/cm^3^)**
LAB	6.57	0.861
Benzene	0.66	0.884
HAB	15.90	0.881

### 3.2 The impact of operating conditions on the density and viscosity of samples

Table 3 reports the viscosity and density measurements obtained under varying experimental parameters, highlighting the conditions that most closely replicate the physical properties of LAB. This analysis offers valuable insights into the adjustments required to fine-tune process variables for achieving LAB characteristics effectively. Statistical analysis (one-way ANOVA, p < 0.05) confirmed significant differences in viscosity across time modules (HBA01–HBA04), with 5-minute irradiation (HBA01, 5.89 cSt) yielding values closest to LAB (6.57 cSt). Similarly, the 4:1 benzene-to-HAB ratio (HBA07, 6.05 cSt) and 10 % AlCl_3_ (HBA14, 5.99 cSt) showed statistically significant alignment with LAB properties (p < 0.05). Temperature effects were also significant, with 20 °C (HBA15, 6.56 cSt) being optimal (p < 0.01).

**Table 3:** Viscosity and density values of samples tested with AlCl_3_ catalyst.

**Sample code**	**Viscosity at 40 °C (cSt)**	**Density at 15^◦^C (g/cm^3^)**
**HBA01**	5.89	0.883
**HBA02**	3.58	0.886
**HBA03**	3.33	0.886
**HBA04**	2.01	0.885
**HBA05**	14.08	0.885
**HBA06**	11.05	0.884
**HBA07**	6.05	0.870
**HBA08**	4.03	0.870
**HBA09**	3.54	0.870
**HBA10**	2.45	0.881
**HBA11**	2.88	0.882
**HBA12**	3.08	0.881
**HBA13**	4.89	0.887
**HBA14**	5.99	0.880
**HBA15**	6.56	0.879
**HBA16**	3.00	0.880
**HBA17**	2.88	0.882
**HBA18**	2.58	0.880

#### 3.2.1 The effect of time on the product’s viscosity and density

Table 3 illustrates the influence of reaction time on viscosity and density within the time module. Density values remained relatively consistent, ranging from 0.883 to 0.885 g/cm^3^ (samples HBA01 to 04), while viscosities showed a marked decline with increased reaction time, decreasing from 5.89 cSt to 2.01 cSt. The optimal ultrasonic irradiation time of 5 min (Sample HBA01) yielded a viscosity of 5.89 cSt (near to LAB viscosity), indicating transalkylation with minimal side reactions.

At 5 min (HBA01), the viscosity (5.89 cSt) and density (0.883 g/cm^3^) closely matched LAB standards, indicating efficient alkyl chain redistribution facilitated by cavitation-induced high-energy microenvironments. Longer irradiation times (10–20 min) reduced viscosity excessively (2.01–3.58 cSt), likely due to over-processing or minor degradation of the alkyl chains. Extended irradiation (10–20 min) resulted in lower viscosities (2.01–3.58 cSt), potentially due to excessive chain cleavage or minor side reactions, such as polymerization, which also correlated with observed color changes. Sample HBA04, subjected to the most extended treatment duration of 20 min, exhibited the lowest viscosity (2.01 cSt), demonstrating that extended reaction times significantly reduce viscosity. Although shorter times (1–4 min) were not tested in this study, they could potentially yield suboptimal conversion rates due to insufficient cavitation energy. These phenomena highlight the critical role of controlled ultrasonic exposure in optimizing reaction outcomes. These results suggest that shorter reaction durations, particularly around 5 min, may be more effective in achieving LAB-like rheological properties while maintaining process efficiency.

#### 3.2.2 The effect of the benzene-to-HAB molar ratio on the product’s viscosity and density

Table 3 demonstrates the effect of varying the benzene-to-HAB molar ratio on the viscosity and density of the samples. Density values ranged from 0.885 to 0.870 g/cm^3^ (samples HBA05 to 09), while viscosities decreased from 14.08 to 3.54 cSt as the benzene-to-HAB ratio increased. Sample HBA09, with a 10:1 ratio, exhibited the lowest viscosity (3.54 cSt); however, this value was below the target viscosity for LAB, indicating overcompensation. Conversely, sample HBA07, at a 4:1 ratio, achieved a viscosity of 6.05 cSt, closely aligning with the target LAB viscosity of 6.57 cSt. These findings suggest that a 4:1 benzene-to-HAB molar ratio is optimal for achieving LAB-like rheological properties, balancing viscosity adjustments, while minimizing deviations from the desired LAB characteristics.

#### 3.2.3 The effect of the catalyst on the product’s viscosity and density

Table 3 highlights the impact of varying AlCl_3_ catalyst concentrations on the viscosities and densities of the samples. Density values ranged from 0.881 to 0.887 g/cm^3^ (samples HBA10 to 14), while viscosities varied between 2.45 and 5.99 cSt. Sample HBA14, with a viscosity of 5.99 cSt, closely matched the target value for LAB. The highest viscosity was observed at the maximum catalyst concentration of 1 %. Conversely, at the lowest catalyst concentration (0.1 %), sample HBA10 exhibited a viscosity of 2.45 cSt, indicating a strong correlation between catalyst concentration and viscosity. Lower catalyst concentrations yielded reduced viscosities, whereas higher concentrations promoted viscosity adjustments toward LAB-like properties. Sample HBA14′s performance underscores the role of increased AlCl_3_ concentration in achieving the desired rheological characteristics, emphasizing its effectiveness in facilitating the HAB-to-LAB conversion process.

#### 3.2.4 The effect of temperature on the product’s viscosity and density

Table 3 illustrates how temperature generally has an inverse relationship with density and viscosity. As the temperature rose from 20 to 80 °C (samples HBA15 to 18), the viscosity dropped from 6.56 to 2.58 cSt, but the density only fluctuated between 0.879 and 0.882 g/cm^3^. After being treated at the lowest temperature (20 °C), sample HBA15 had a viscosity of 6.56 cSt, which was reasonably close to the target viscosity of 6.57 cSt for LAB. It suggests that obtaining viscosities similar to those of LAB is best achieved at lower temperatures, whereas it significantly decreases at higher temperatures. Therefore, the ideal temperature for adjusting sample characteristics to match those of LAB is 20 °C.

### 3.3 Sample appearance test

The appearance test was conducted during the project to evaluate the similarity of various samples from HAB to LAB conversion under ultrasonic treatment with an AlCl_3_ catalyst. The four experimental variables tested were time, benzene-to-HAB ratio, catalyst concentration, and temperature, as shown in Table 4. Each module required altering one factor while keeping the other variables constant. In the **time module**, with benzene set at 10 g, temperature at 20 °C, and catalyst at 10 g, sample HBA01 showed an appearance close to LAB after 5 min. However, the appearance became less similar to LAB as the reaction time increased. The change in solution color with increased irradiation time (5 to 20 min) may be attributed to minor side reactions, such as the formation of colored by-products or partial degradation of alkyl chains due to prolonged exposure to ultrasonic cavitation. At 5 min (HBA01), the product closely resembled LAB in appearance, suggesting optimal conversion with minimal side reactions. Longer durations (e.g., HBA04 at 20 min) likely induced trace polymerization or oxidation, altering the color.

**Table 4:** Appearance of samples under different time, benzene-to-HAB ratio, catalyst percentage, and temperature modules.

**Sample code**	**Time module samples**	**Sample code**	**Benzene to HAB module samples**	**Sample code**	**Catalyst module samples**	**Sample code**	**Temperature module samples**
HBA01		HBA05		HBA10		HBA15	
HBA02		HBA06		HBA11		HBA16	
HBA03		HBA07		HBA12		HBA17	
HBA04		HBA08		HBA13		HBA18	
		HBA09		HBA14			

In brief, for the **benzene-to-HAB ratio module**, under fixed conditions of 20 °C, 5 min, and 1 g of catalyst, sample HBA07, with a 4:1 ratio (10 g of benzene), had the closest appearance to LAB, while other ratios deviated further. In the **catalyst concentration module**, with conditions of 20 °C, 5 min, and 10 g of benzene, sample HBA14, at a 10 % catalyst concentration, most closely resembled LAB, indicating that increasing the catalyst concentration improved the match. For the **temperature module**, under the same conditions of 5 min, 10 g of benzene, and 1 g of catalyst, sample HBA15 showed the most LAB-like appearance when the temperature was maintained at 20 °C. It was noted that higher temperatures resulted in products that deviated further from LAB.

The experimental conditions that yielded a product with an appearance most comparable to linear alkylbenzene (LAB) were identified as a 5-minute reaction time, a 4:1 benzene-to-HAB molar ratio, a 10 % catalyst concentration, and a reaction temperature of 20 °C. Under these optimized parameters, the maximum conversion of HAB to LAB was achieved, as observed in samples HBA01, HBA07, HBA14, and HBA15 since they have the same conditions. Among these, sample HBA01 was selected—along with the reference compounds HAB and LAB—for further characterization using Fourier Transform Infrared Spectroscopy (FTIR) and for determination of the aniline point and flash point.

### 3.4 Fourier transform infrared spectroscopy.

**Fig. 2:**
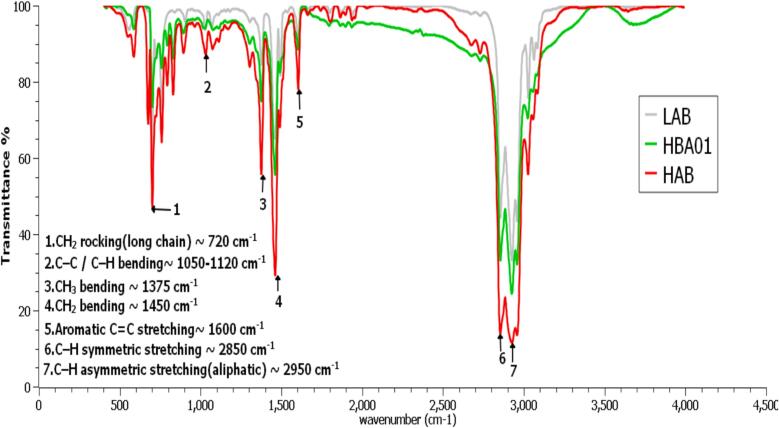
FTIR spectra of linear alkylbenzene (LAB), ultrasonic-treated sample (HBA01), and heavy alkylbenzene (HAB).

FTIR spectroscopy was employed to monitor the structural transformation from HAB to LAB. As illustrated in Fig. 2, the HAB spectrum displays characteristic aromatic features, such as the C=C stretching at ∼ 1600 cm^−1^ and CH_2_ rocking at ∼ 720 cm^−1^. In contrast, the LAB spectrum shows prominent aliphatic peaks around ∼ 2950 and ∼ 2850 cm^−1^, indicating the presence of long linear alkyl chains. The spectra also demonstrate the gradual transformation of HAB into LAB through the intermediate sample HBA01. The characteristic absorption bands at ∼ 2950 and 2850 cm^−1^ (C–H stretching), 1450 cm^−1^ (CH_2_ bending), and 720 cm^−1^ (CH_2_ rocking) become progressively sharper and more intense from HAB to HBA01 and LAB, indicating reduced branching and increased formation of long, linear alkyl chains. These spectral changes confirm that ultrasonic treatment effectively promotes the structural conversion of heavy alkylbenzene toward the linear alkylbenzene configuration.

Table 5 provides quantitative transmittance values that highlight the progression of the transformation. For example, transmittance at ∼ 2950 cm^−1^ increases from 13.5 % in HAB to 32 % in HBA01, reflecting a reduction in chain branching. Additionally, the CH_2_ rocking band at ∼ 720 cm^−1^ rises significantly in HBA01 (73.2 %) compared to HAB (46.8 %), indicating the formation of long straight chains typical of LAB. These observations demonstrate that HBA01 is not merely a transitional sample, but a structurally advanced intermediate that reflects a meaningful shift toward LAB.

**Table 5:** Comparative FTIR transmittance values at characteristic peaks for HAB, HBA01, and LAB samples indicating structural transformation.

**Wavenumber (cm**^−^**^1^)**	**Vibrational Mode**	**HAB (%)**	**HBA01 (%)**	**LAB (%)**	**Scientific Comment**
**2950**	C–H asymmetric stretching (CH_3_, CH_2_)	13.5	32	43.4	Transmittance increases progressively, suggesting transformation toward LAB through reduced branching.
**2850**	C–H symmetric stretching (CH_3_, CH_2_)	13.8	32.3	44	Confirms similar trend, supporting shift toward linear alkyl chain configuration.
**1600**	Aromatic C=C stretching	78	88.3	91	Higher transmittance indicates reduced substitution on the aromatic ring and increased LAB-like purity.
**1450**	CH_2_ bending	29.2	55.5	65	Stronger CH_2_ bending in HBA01 and LAB suggests longer, less branched alkyl chains.
**1375**	CH_3_ bending	55.7	74.7	84.4	Increase in CH_3_ bending intensity reflects cleaner chain formation and less tertiary carbon content.
**1120–1050**	C–C stretching or C–H bending in-plane bending	88.6	93.7	94.4	Small increase implies subtle structural refinement toward LAB-type chain arrangement.
**720**	CH_2_ rocking (long straight-chain indicator)	46.8	73.2	59.4	The higher transmittance in HBA01 suggests enhanced formation of straight-chain alkyl structures, showing more defined transformation toward LAB.

### 3.5 NMR spectroscopic analysis

#### ^3.5.1^ ^1^H NMR analysis (500 MHz, CDCl_3_)

**Fig. 3:**
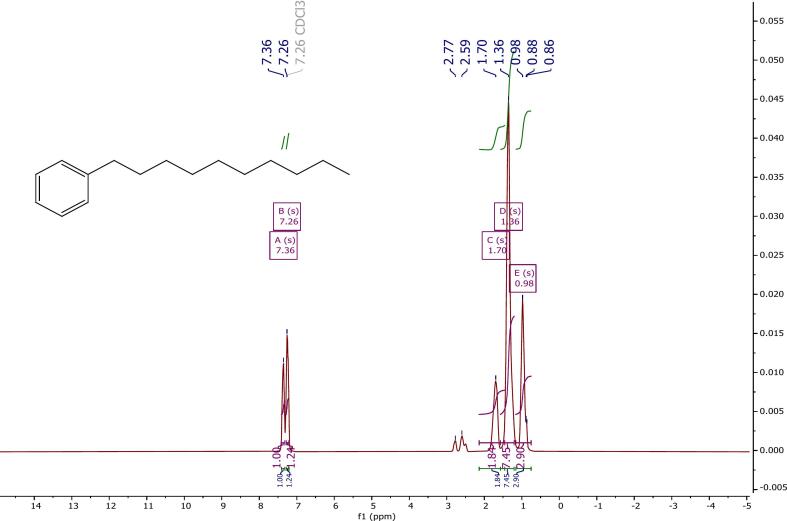
^1^H NMR spectrum of the ultrasonic-treated sample HBA01 (CDCl_3_, 500 MHz).

The ^1^H NMR spectrum of the synthesized product was recorded using a 500 MHz spectrometer with CDCl_3_ as the solvent (Fig. 3). The spectrum provides clear evidence of alkyl chain substitution on the aromatic ring, indicating monosubstitution. This conclusion is supported by the presence of two singlet peaks in the range of 7.36–7.26 ppm, corresponding to the aromatic protons of a substituted benzene ring. In addition, a distinct signal appearing between 2.77–2.59 ppm is attributed to the benzylic (–CH_2_–Ar) protons, further confirming that an alkyl chain is directly attached to the aromatic methine proton. These spectral features collectively verify the successful formation of the desired linear alkylbenzene (LAB) structure through the catalytic transalkylation process. The following signals are present in the aliphatic region:

• Methylene protons benzylic to the boron are at *δ* 1.70 ppm with a multiplicity of s (2H).
• Internal methylene groups have been assigned *δ* 1.36 (s, 7H).
• Terminal methyl groups have a range of *δ* 0.98 (s, 3H).

These signals confirm the successful formation of a long, linear alkyl chain attached to a mono-substituted aromatic ring, consistent with the expected structure of the transformed HBA01 sample.

Given that the ensuing peaks exhibit the expected proton distribution for monoalkylated aromatic compounds, it is reasonable to conclude that the obtained product corresponds to linear alkylbenzene (LAB). The most significant chemical shifts and corresponding proton assignments are summarized in Table 6. It is noteworthy that all detected signals appeared as singlets, which may indicate equivalent proton environments or limited coupling resolution under the applied measurement conditions. Furthermore, subtle variations related to multiplicity or chain-end effects might not be fully distinguishable within the recorded spectrum, possibly due to the overlap of closely spaced signals or the influence of the solvent environment.

**Table 6:** Key ^1^H NMR Chemical Shifts and Assignments for HBA01 (CDCl_3_, 500 MHz).

**Chemical Shift (*δ*, ppm)**	**Multiplicity**	**Integration**	**Assignment**	**Interpretation**
7.36	s	∼1H	Aromatic proton	Mono-substituted benzene ring
7.26	s	∼1H	Aromatic proton	
2.77	s	∼1H	Benzylic methylene (–CH_2_–Ar)	Indicates direct alkylation at aromatic ring
2.59	s	∼1H	Benzylic methylene (–CH_2_–Ar)	
1.70	s	∼2H	α-Methylene (next to benzylic CH_2_)	
1.36	s	∼7H	Internal methylene (–CH_2_-)	Suggests long unbranched chain
0.98	s	∼3H	Terminal methyl (–CH_3_)	Chain end

#### ^3.5.2^ ^13^C NMR analysis (126 MHz, CDCl_3_)

**Fig. 4:**
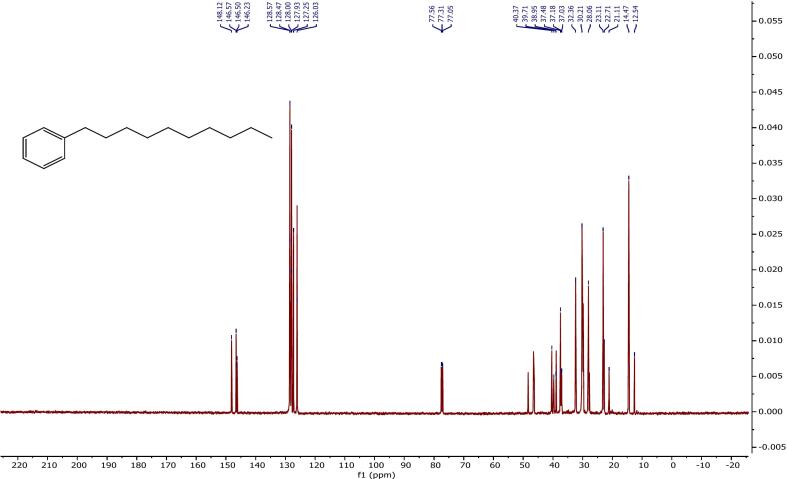
^13^C NMR spectrum of the ultrasonic-treated sample HBA01 (CDCl_3_, 125 MHz).

Fig. 4 depicts the ^13^C NMR spectrum of the product that was recorded at 126 MHz using CDCl_3_ as solvent. The spectrum provides crucial information regarding the carbon environments present in the molecule like aromatic, benzylic, aliphatic, and terminal methyl carbons.

• Aromatic Carbon Signals (*δ* 148.12–126.03 ppm):

The signals observed at *δ* 148.12, 146.57, 146.50, and 146.23 ppm are due to non-protonated quaternary aromatic carbons, and the signals observed at *δ* 128.57, 128.47, 128.00, 127.93, 127.25, and 126.03 ppm are due to protonated aromatic carbons of the benzene ring. They are in accordance with the characteristics of a mono-substituted aromatic ring.

• Benzylic Carbon Signal (δ ∼ 40–38 ppm):

The resonances at *δ* 40.37, 39.71, and 38.95 ppm are due to benzylic methylene carbon atoms (–CH_2_–Ar), which are significant evidence for an alkyl chain directly attached with the aromatic ring.

• Aliphatic Methylene Carbon signals (δ ∼ 37–22 ppm):

Signals at *δ* 37.48, 37.18, and 37.03 ppm, together with *δ* 32.36, 30.21, 28.06, 23.11, 22.71, and 21.11 ppm, are attributed to methylene (–CH_2_-) groups of the linear alkyl chain.

• Terminal methyl carbon signals (*δ* 14.47 and 12.54 ppm):

The peaks seen verify the existence of terminal methyl (–CH_3_) groups, which are characteristic of a long alkyl chain.

• Solvent Residual Peaks:

As expected, peaks at *δ* 77.56, 77.31, and 77.05 ppm are from residual CDCl_3_ solvent. These observed chemical shifts agree with the reference data obtained from the Spectral Database for Organic Compounds (SDBS), thereby corroborating the anticipated structural characteristics of a LAB. The minor deviations observed in the aliphatic region can be attributed to slight variations in the chain length or differences in instrument conditions. Table 7 provides a summary of the significant ^13^C NMR signals and their respective assignments.

**Table 7:** ^13^C NMR Chemical shifts and carbon assignments for HBA01 sample.

**Chemical Shift δ (ppm)**	**Carbon Type**	**Assignment**
148.12, 146.57, 146.50, 146.23	Quaternary aromatic carbons	Non-protonated carbons on benzene ring
128.57, 128.47, 128.00, 127.93, 127.25, 126.03	Protonated aromatic carbons	Aromatic carbons bonded to protons
40.37, 39.71, 38.95	Benzylic methylene carbons	–CH_2_- group directly attached to aromatic ring
37.48, 37.18, 37.03, 32.36, 30.21, 28.06, 23.11, 22.71, 21.11	Aliphatic methylene carbons	Internal –CH_2_- groups of the alkyl chain
14.47, 12.54	Terminal methyl carbons	–CH_3_ groups at the end of the alkyl chain

**Fig. 5:**
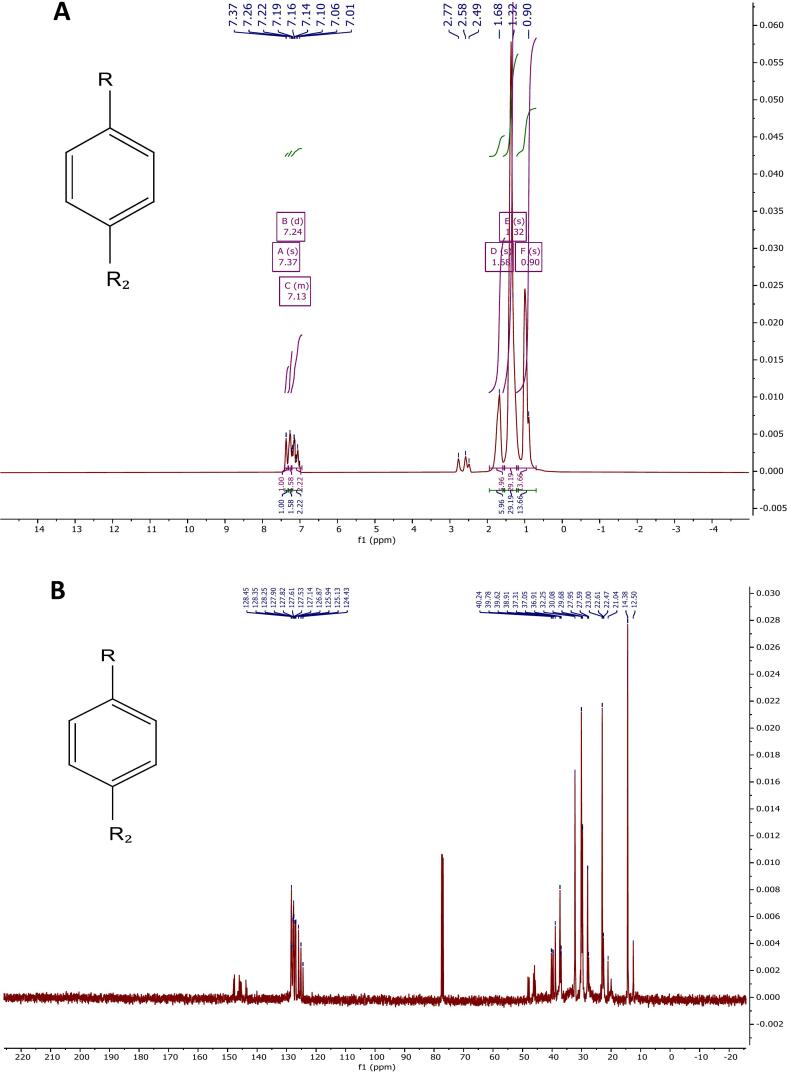
A. ^1^H NMR spectrum of HAB, B. ^13^C NMR spectrum of HAB.

To confirm the structural transformation from the initial compound (HAB) to the synthesized product (HBA01), a comparative analysis of their ^1^H and ^13^C NMR spectra was carried out. Fig. 5 shows both the ^1^H NMR and ^13^C NMR spectra of HAB, compared with the ^1^H NMR and ^13^C NMR spectrum (Fig. 3, Fig. 4), the appearance of additional peaks in the aliphatic region (*δ* 0.8–2.5 ppm) and the reduction of aromatic proton signals (*δ* 7.0–7.5 ppm) in HBA01 relative to HAB indicate the formation of a linear alkyl chain bonded to the benzene ring, confirming the alkylation process. Correspondingly, the ^13^C NMR spectrum of HBA01 exhibits new aliphatic carbon peaks (*δ* 14–40 ppm) while retaining the aromatic carbon signals (*δ* 120–140 ppm), signifying successful alkylbenzene formation. The ^1^H NMR spectra further reveal distinct variations in the aliphatic and benzylic regions. For HAB, the upfield signals (*δ* 0.8–1.5 ppm) denote the presence of a long alkyl chain, whereas in HBA01 these signals are significantly reduced, implying chain modification or shortening during the reaction. This observation supports the conversion of HAB to LAB, consistent with the AlCl_3_-catalyzed transalkylation under ultrasonic irradiation.

In the aromatic region (*δ* 6.5–8.0 ppm), both spectra display multiplets typical of aromatic protons, though minor chemical shift changes suggest variations in the substitution pattern on the ring. The benzylic methylene protons (–CH_2_–Ar) appear in both spectra but show a slight downfield shift in HBA01, confirming monoalkylation. The ^13^C NMR data provide further support, as HBA01 presents fewer aliphatic carbon signals and subtle aromatic carbon shifts, consistent with electronic environment modification following monoalkyl substitution. Overall, the combined spectroscopic evidence verifies that the synthesized product corresponds to linear alkylbenzene (LAB), confirming the successful transformation of HAB.

### 3.6 Gas chromatography

Here, GC analysis of the synthesized sample HBA01 was conducted through a **Bruker Scion 436-GC** gas chromatograph. The resulting chromatogram contained multiple peaks for different LAB isomers with minimal occurrence of non-target compounds. Quantified percentage of area for each of the compounds detected is tabulated below in Table 8.

**Table 8:** GC analysis of the HBA01 sample.

**Name**	**Area [v.min]**	**Quantity [% area]**	**MWT**	**NONLABHAB**
NP	499.0	0.24	240.09	7.68
Total LAB	189121.7	92.08		
2.Ph	55886.1	27.21		
Ph C9	565.3	0.28		
Ph C10	27064.5	13.18		
Ph C11	63914.0	31.12		
Ph C12	57763.0	28.12		
Ph C13	39716.2	19.34		
Ph C14	98.7	0.05		

The GC result shows that most of the product is in the LAB range (C10–C13), which takes up more than 91 % of the total area. The most abundant isomers in the product were Ph C11 (31.12 %) and Ph C12 (28.12 %), which are normally the most desirable fractions in commercial LAB because they degrade easily and have good behavior in detergent formulations. The virtual lack of higher-chain (C14) and non-alkylated fragments like NP (0.24 %) shows that the process is selective. The very small amount of pH C9 (0.28 %) can be attributed to trace amounts of under-alkylated species. These findings agree with the FTIR and NMR findings. They reveal that the use of ultrasound in the presence of AlCl_3_ as a catalyst was effective to synthesize LAB as per industrial specifications. The composition of the product shows high product purity and conversion efficiency, confirming that the process was successful.

### 3.7 Aniline point and flash point of the samples

Table 9 presents the aniline point test results for multiple samples, including LAB. The LAB sample exhibited an aniline point of 12 °C, whereas samples HBA01, HBA07, HBA14, and HBA15 had aniline points ranging from 16 °C to 22 °C. This range is closely aligned with that of the LAB sample, suggesting a successful conversion of HAB into LAB. The similarity in aniline points between the converted samples and LAB indicates that the conversion process was effective, as reflected in the properties of the resulting samples. Table 9 presents the flash point test results for LAB, HBA01, HBA07, HBA14, and HBA15. The LAB sample has a flash point of 120 °C, whereas samples HBA01, HBA07, HBA14, and HBA15 exhibit flash points ranging from 115 °C to 117 °C, closely matching that of LAB. The similarity in flash points confirms a successful conversion from HAB to LAB, indicating that the transformed samples possess flammability properties comparable to those of LAB. This similarity further reinforces that the physical and chemical properties of the samples are consistent with those of LAB, validating the effectiveness of the conversion process. In conclusion, the flash point results clearly demonstrate the successful conversion of HAB into LAB, as evidenced by flash points nearly identical to that of LAB. This provides strong evidence of the effectiveness of the methods employed in this study.

**Table 9:** The results of the performed flash point and aniline point tests.

**Sample Code**	**Flash point (°C)**	**Aniline point (°C)**
**LAB**	120	12
**HBA01**	115	22
**HBA07**	117	16
**HBA14**	117	22
**HBA15**	117	22

### 3.8 Proposed mechanism of the transalkylation reaction

The transformation of HAB into LAB is commonly described as a Friedel–Crafts alkylation process involving the redistribution of alkyl groups among aromatic compounds. The proposed reaction mechanism is based on the interaction between benzene and a substituted benzene derivative, where R^1^ and R^2^ denote alkyl substituents. A Lewis acid catalyst, typically AlCl_3_, facilitates this transformation by polarizing or abstracting a chloride ion from the substrate, resulting in the generation of an electrophilic species such as a carbocation [22,23]. This electrophilic intermediate can then react with a benzene ring, leading to alkyl group transfer and the formation of a new substituted benzene molecule (i.e., LAB). A generalized depiction of this mechanism is shown below (equation 1):(1)
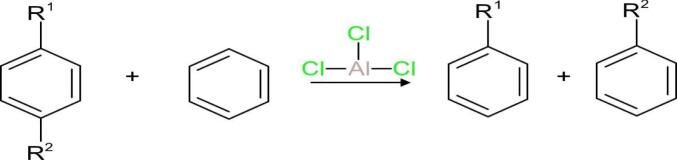


This pathway aligns with previously reported Lewis acid-catalyzed transalkylation mechanisms, including those using solid acid catalysts such as zeolites [24]. In the initial activation step, the interaction between AlCl_3_ and the alkyl group on HAB is proposed to lead to cleavage of a C–H bond and the formation of a carbocationic intermediate. Equation 2 illustrates this proposed activation:(2)
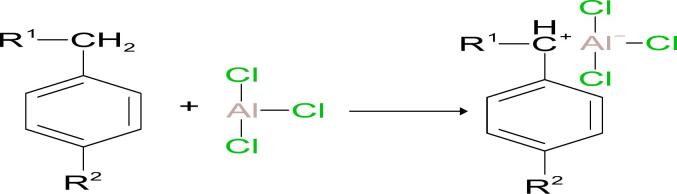


Incorporating ultrasonic energy into the reaction system introduces additional effects that may enhance the process. Ultrasonic cavitation—defined as the formation, growth, and implosive collapse of microbubbles in the liquid phase—generates localized high-temperature (up to several thousand Kelvin) and high-pressure (hundreds of atmospheres) zones [17]. These extreme, transient conditions are known to facilitate bond cleavage and the formation of reactive intermediates in sonochemical systems [25,26]. The possible contributions of ultrasonic cavitation in this context include:

• Improving the dispersion and reactivity of AlCl_3_ by increasing its effective surface area.
• Enhancing the rate and stability of carbocation formation under localized energetic conditions.
• Disrupting intermolecular forces in HAB, thereby exposing more reactive sites.

The next step involves the attack of the benzene ring on the carbocation, as shown in the following reaction (Equation 3):(3)
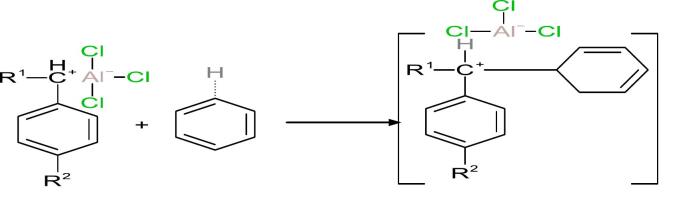


This nucleophilic substitution reaction is stabilized by resonance effects within the aromatic ring, leading to the formation of the linear alkylbenzene (LAB) product (Equation 4).(4)
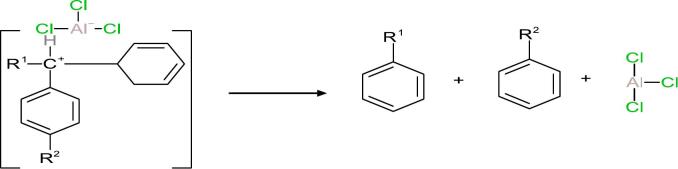


Ultrasonic agitation also aids in maintaining a uniform reaction environment, minimizing diffusion limitations and potentially suppressing undesired side reactions such as polyalkylation. It also may further assist in sustaining catalyst activity by preventing aggregation and promoting recyclability. Together, these effects contribute to an efficient and potentially greener reaction system. To sum up, the results validate that HAB can be effectively converted to LAB by ultrasonic-induced transalkylation. In this method, HAB is used as a catalyst in the presence of ultrasonic vibrations. Compared to traditional techniques, it significantly improves reaction kinetics and energy efficiency. These enhancements demonstrate their potential for use in industrial settings. The use of an ultrasonic bath at 37 kHz and 150 W generates sufficient cavitation to enhance transalkylation without significant sonolysis of the benzene solvent.

Sonolysis, the chemical decomposition of solvents under ultrasonic irradiation, is minimal in anhydrous organic solvents like benzene due to their high vapor pressure and low polarity, which reduce radical formation compared to aqueous systems[17,18]. This ensures that the primary effect of ultrasound is mechanical (e.g., improved mixing and catalyst dispersion) rather than chemical degradation of the solvent. The ultrasonic-induced transalkylation process demonstrates superior efficiency compared to traditional methods, as evidenced by the high LAB purity (91 % C10–C13 isomers) achieved in 5 min at 20 °C. This contrasts with studies like Ghadiri et al. [9], which required 225 °C for 80 % conversion, highlighting the energy-saving potential of ultrasound. The cavitation-induced microenvironments enhance AlCl_3_ dispersion and mass transfer, reducing reaction times and side reactions. However, the color changes observed at longer irradiation times (Table 4) suggest minor side reactions, which could be mitigated by optimizing ultrasound parameters.

### 3.9 Role of ultrasound in transalkylation mechanism

The application of ultrasonic energy significantly enhances the transalkylation process by inducing acoustic cavitation, which creates transient high-energy microenvironments. During cavitation, the collapse of microbubbles generates localized temperatures of several thousand Kelvin and pressures of hundreds of atmospheres, facilitating the cleavage of C–H and C–C bonds in HAB[17]. This promotes the formation of carbocation intermediates, which are critical for the Friedel-Crafts transalkylation reaction catalyzed by AlCl_3_. Additionally, ultrasound improves the dispersion of AlCl_3_, increasing its effective surface area and interaction with reactants. The mechanical effects of cavitation, such as microstreaming and jet formation, enhance mass transfer and expose fresh catalytic sites, reducing reaction times and energy requirements[18]. Compared to traditional methods, this approach minimizes side reactions (e.g., polyalkylation) by maintaining a uniform reaction environment and preventing catalyst aggregation [27]. The enhanced kinetics and efficiency observed in our experiments, particularly at 5 min of ultrasonic treatment (e.g., HBA01 with 5.89 cSt viscosity), underscore the synergistic effect of ultrasound and AlCl_3_ catalysis in achieving high linear alkylbenzene (LAB) purity (91 % C10–C13 isomers) under sustainable conditions.

### 3.10 Comparison between conventional and ultrasound transalkylation

Compared to conventional transalkylation methods using zeolites or other solid acid catalysts, the ultrasound-induced approach offers clear advantages. Although solid acid catalysts such as zeolites are environmentally friendly, their slower reaction rates and higher operational temperatures often limit their industrial scalability. It is worth noting that initial experiments using zeolites and AlCl_3_ under standard (non-ultrasonic) conditions were also carried out, but they did not yield promising results. In contrast, using HAB under ultrasonic conditions achieves comparable or even superior performance while reducing both thermal and operational demands. Furthermore, the resulting LAB meets industrial standards in terms of purity, viscosity, and density, validating the feasibility of this method for large-scale applications. As shown in Table 10, the ultrasonic-treated samples exhibit viscosity and density values that are nearly identical to those of standard LAB. Additionally, the appearance of the product under ultrasonic treatment closely resembles that of LAB, unlike the outcome of the traditional method. Compared to Ghadiri et al.[9], who achieved 80 % HAB conversion at 225 °C, our ultrasonic-induced method operates at 20 °C, reducing energy consumption while maintaining high LAB purity. Tsai et al.[20] reported high benzene purity but required higher temperatures and longer reaction times. The integration of ultrasound in our study enhances catalyst efficiency and reaction kinetics, offering a greener alternative with comparable or superior performance[5,18]. Control experiments under traditional (non-ultrasonic) conditions were conducted at 100 °C for 60 min using 10 g HAB, 4:1 benzene-to-HAB ratio, and 1 g AlCl_3_, yielding lower viscosities (3.60–4.56 cSt, Table 10) compared to ultrasonic conditions (20 °C, 5 min), highlighting the efficiency of ultrasound in achieving LAB standards.

**Table 10:** Comparison between traditional and ultrasonic-induced methods in LAB production.

**Traditional method**					**Ultrasound method**				
**Sample code**	**Viscosity At 40◦ C(cSt)**	**Density at 15 ◦C (g/cm3)**	**Appearance**	**Conditions**	**Sample code**	**Viscosity At 40◦ C(cSt)**	**Density at 15◦C (g/cm3)**	**Appearance**	**Conditions**
**Sample 4**	3.60	0.886		100 °C, 60 min	**HBA01**	5.89	0.883		20 °C, 5 min
**Sample 5**	4.01	0.882		100 °C, 60 min	**HBA07**	6.05	0.870		20 °C, 5 min
**Sample 6**	4.56	0.884		100 °C, 60 min	**HBA14**	5.99	0.880		20 °C, 5 min
					**HBA15**	6.56	0.879		20 °C, 5 min

#### 3.10.1 Economic estimation

The ultrasonic-induced method reduces energy costs by operating at 20 °C compared to traditional methods requiring 200–600 °C (e.g., Ghadiri et al. [9], Tsai et al. [20]). Assuming a 50 % reduction in energy consumption (based on lower temperature and 5-minute reaction time vs. hours for conventional methods), and with AlCl_3_ recovery at ∼ 90 %, the process could lower operational costs by 30–40 %. A detailed cost-benefit analysis, including equipment and catalyst recycling costs, is recommended for industrial-scale validation [5].

A key advantage of this method lies in its alignment with green chemistry principles, particularly in terms of reduced energy input, lower operating temperatures, and shorter reaction times, all of which contribute to a lower environmental footprint. Although HAB is known to be corrosive, its use in this process is strictly controlled under anhydrous conditions, and no aqueous quenching is applied. After the reaction, AlCl_3_ is recovered by simple filtration, minimizing chemical waste and preventing the formation of acidic aqueous byproducts. After the reaction, AlCl_3_ was recovered by simple filtration with an estimated recovery rate of approximately 90 %. The recovered catalyst was reused for up to three cycles, maintaining approximately 85 % of its initial activity based on viscosity measurements of subsequent reactions (e.g., 5.89 cSt for HBA01 in the first cycle vs. 5.60–5.75 cSt in subsequent cycles). The effectiveness of the recovery process was confirmed by the consistency of LAB purity (91 % C10–C13 isomers) across reuse cycles, indicating minimal loss of catalytic performance. Further studies are needed to assess the long-term stability of AlCl_3_ under repeated ultrasonic conditions to optimize its reuse for industrial applications [22]. Furthermore, the process operates efficiently with just 10 wt% of AlCl_3_, reducing the environmental burden associated with catalyst usage. The ability to recycle the catalyst and convert HAB—a low-value by-product—into high-demand LAB-like compounds also supports the principles of waste valorization and circular economy, promoting a more sustainable chemical process.

### 3.11 Limitations and recommendations

While this study demonstrates the efficacy of ultrasonic-induced transalkylation, certain limitations warrant further investigation. The effects of ultrasound power, frequency, and atmospheric gas (e.g., inert vs. ambient air) were not systematically explored, which could influence cavitation efficiency. Additionally, the economic feasibility of scaling this process and the long-term stability of AlCl_3_ under repeated ultrasonic cycles require further study. Future research should focus on optimizing ultrasound parameters (e.g., frequencies beyond 37 kHz), testing shorter irradiation times (1–4 min), and conducting a detailed cost-benefit analysis to assess industrial applicability. These advancements could further enhance the sustainability and efficiency of LAB production. We recommend further optimization of ultrasonic parameters, including testing frequencies beyond 37 kHz and irradiation times below 5 min to minimize energy input. A comprehensive economic analysis and pilot-scale studies are advised to validate industrial scalability, ensuring alignment with sustainable production goals.

## 4 Conclusions

This study effectively demonstrated the efficiency of ultrasonic-induced transalkylation in converting HAB into LAB, a key component in the detergent industry. By transforming a low-value by-product into a valuable industrial feedstock while adhering to sustainable chemical principles, this method provides a scalable and environmentally responsible alternative for LAB production. It was demonstrated that the thechnique significantly shortened reaction times while increasing overall conversion efficiency by adjusting parameters like temperature, benzene-to-HAB ratio, catalyst concentration, and ultrasonic treatment duration. Comprehensive characterization via FTIR, GC, and both ^1^H and ^13^C NMR spectroscopy confirmed the structural transformation from branched or high-molecular-weight alkyl chains in HAB to linear monoalkylated benzene derivatives characteristic of LAB. Also, GC analysis showed that over 91 % of the product fell within the C10–C13 LAB range, while NMR and FTIR data further confirmed the successful alkyl chain redistribution. The integration of ultrasonic energy significantly enhanced mass transfer, enabling effective conversion under milder conditions. This not only improves process efficiency but also aligns with green chemistry principles by minimizing thermal and environmental burdens. Although AlCl_3_ is a conventional Lewis acid catalyst, its controlled application under sonochemical conditions offers an effective and scalable alternative to traditional high-temperature routes or heterogeneous systems. These results highlight the potential of ultrasonic technology to replace conventional LAB production methods by offering a more environmentally friendly and energy-efficient alternative.

## CRediT authorship contribution statement

**Abdallah S. Elgharbawy:** Writing – review & editing, Validation, Supervision, Conceptualization. **Wagih A. Sadik:** . **Mosaad A. Kasaby:** Funding acquisition, Formal analysis. **Ahmed A. Ghazy:** Writing orignal and resources.

## Declaration of competing interest

The authors declare that they have no known competing financial interests or personal relationships that could have appeared to influence the work reported in this paper.
